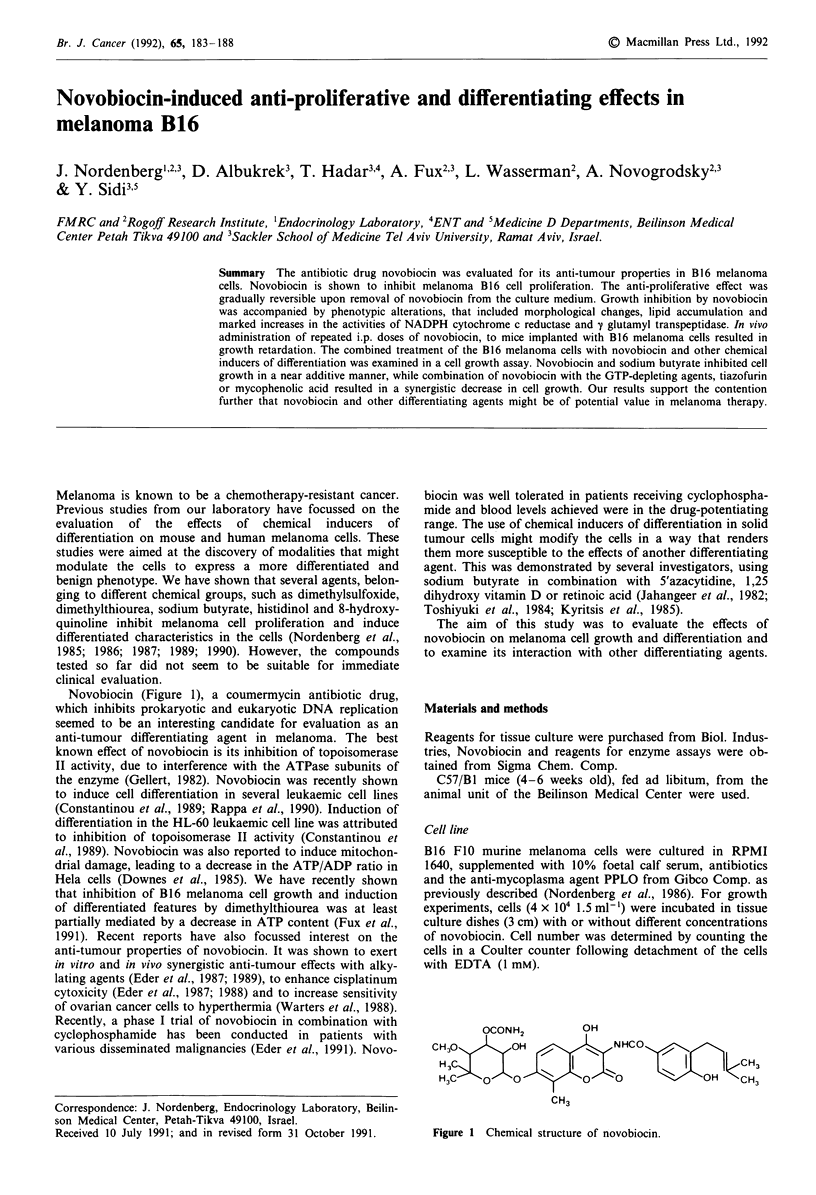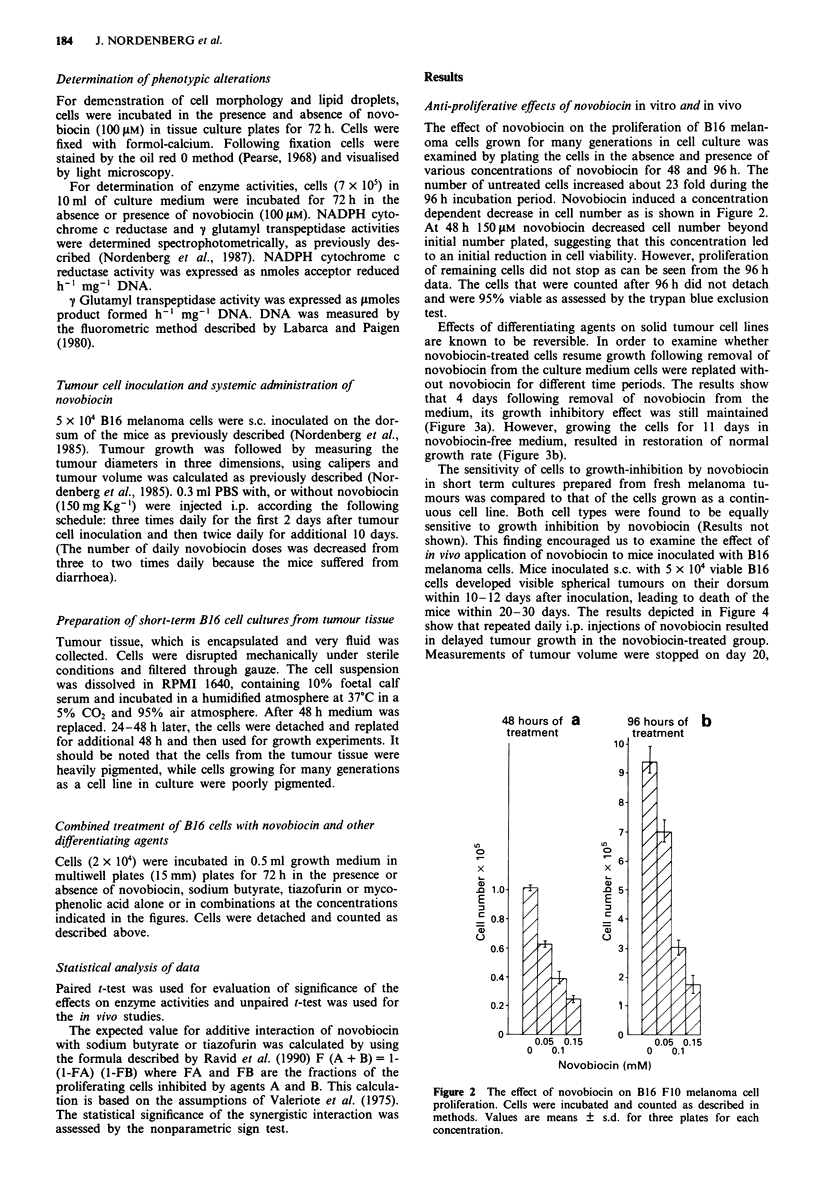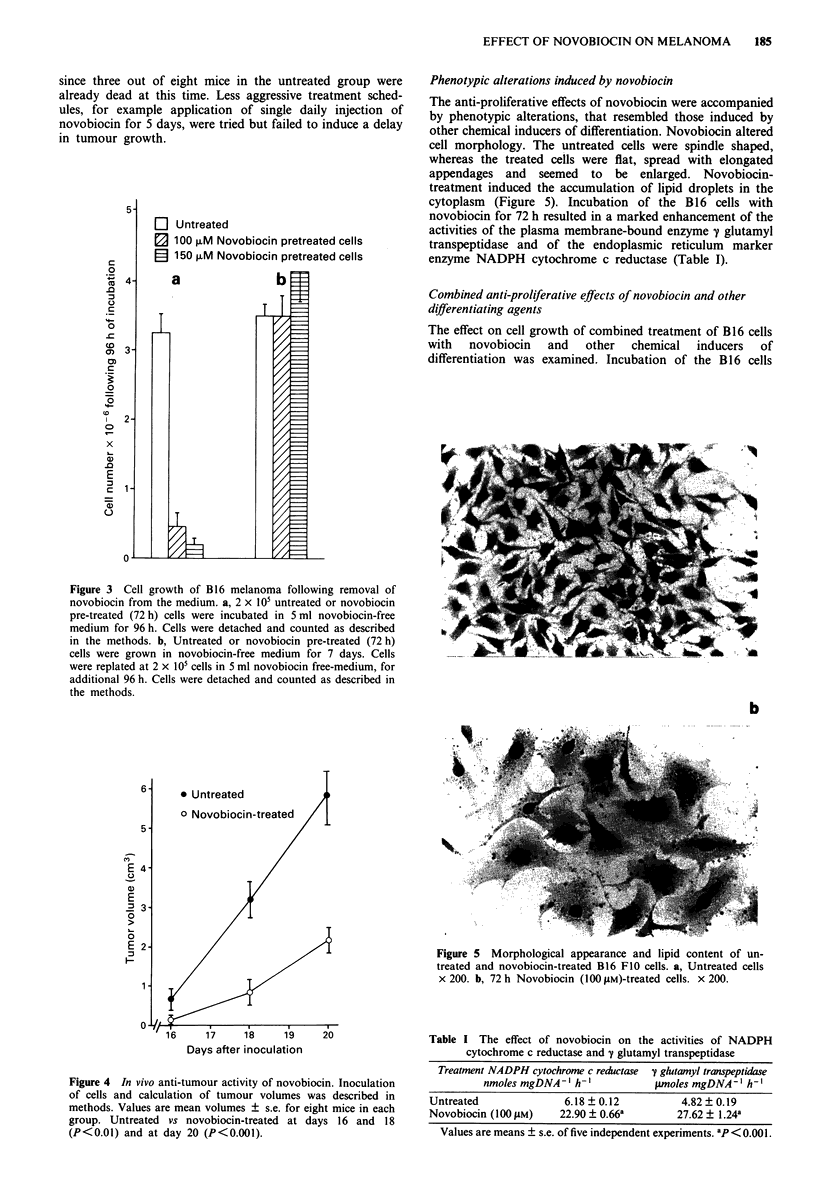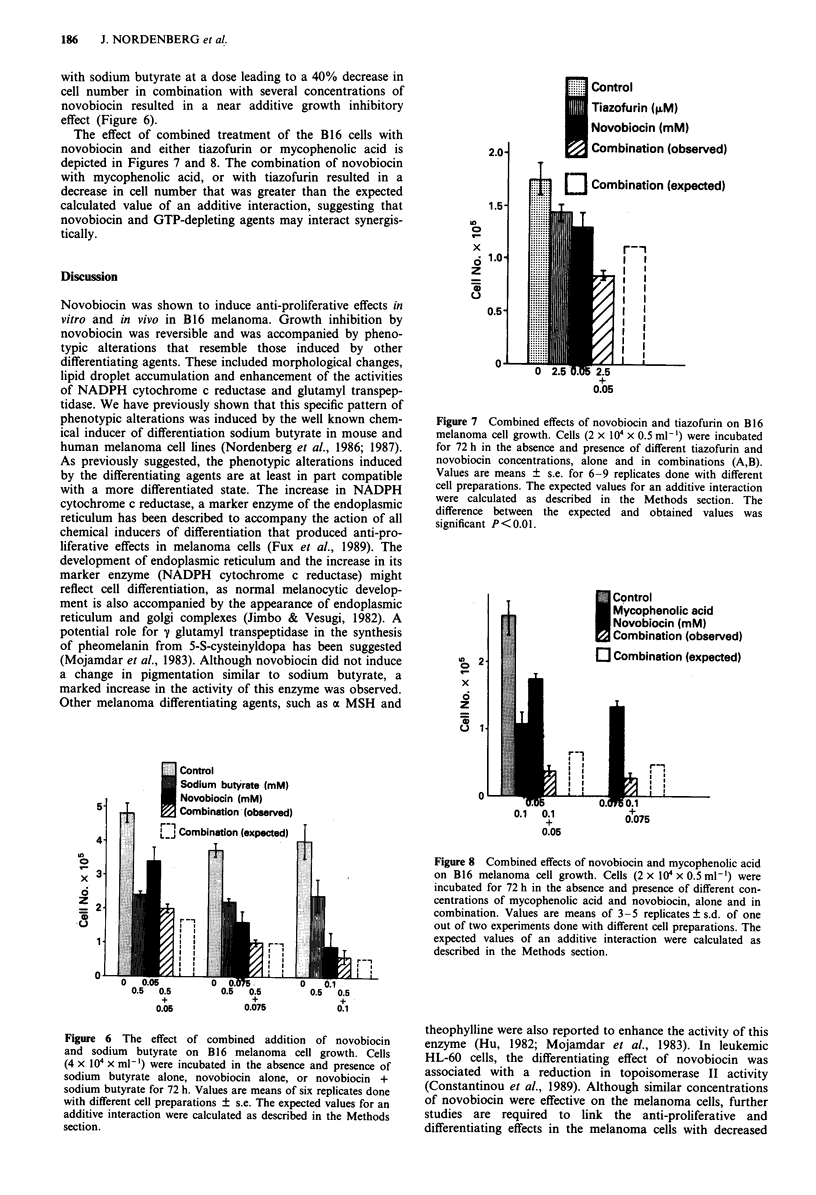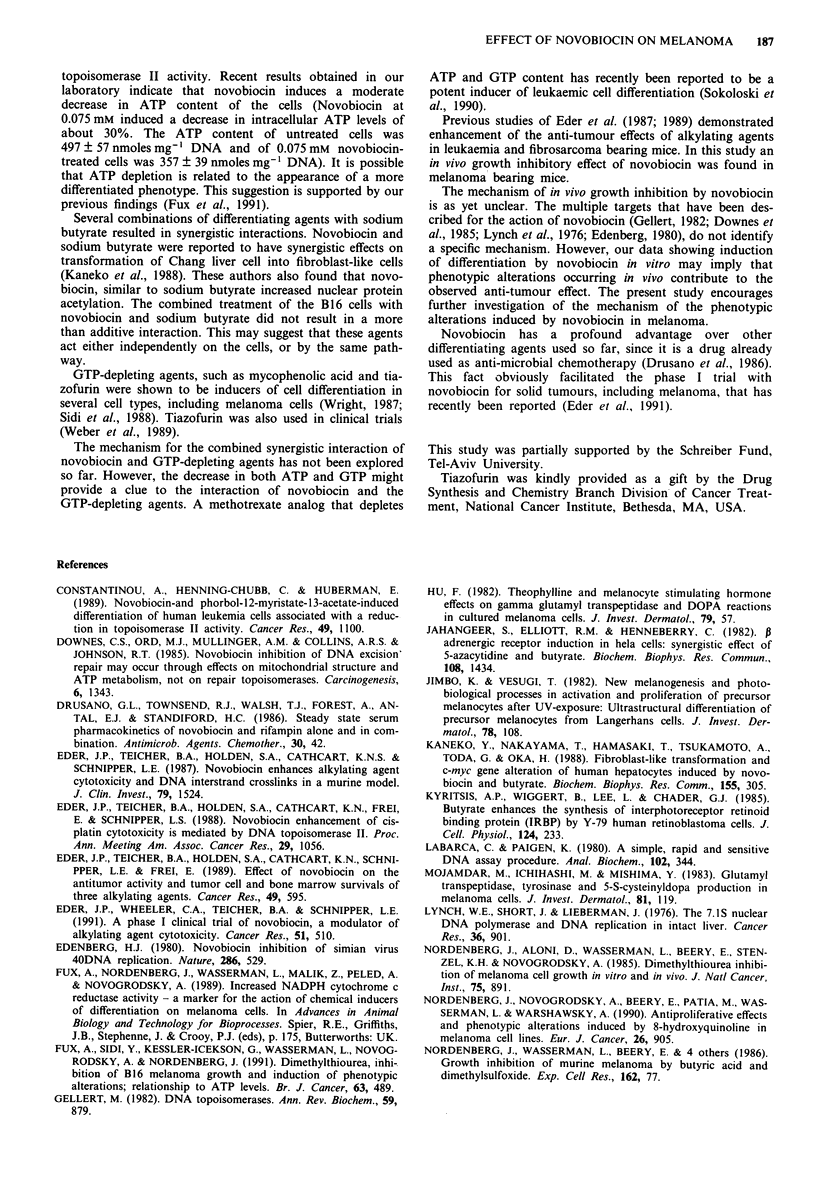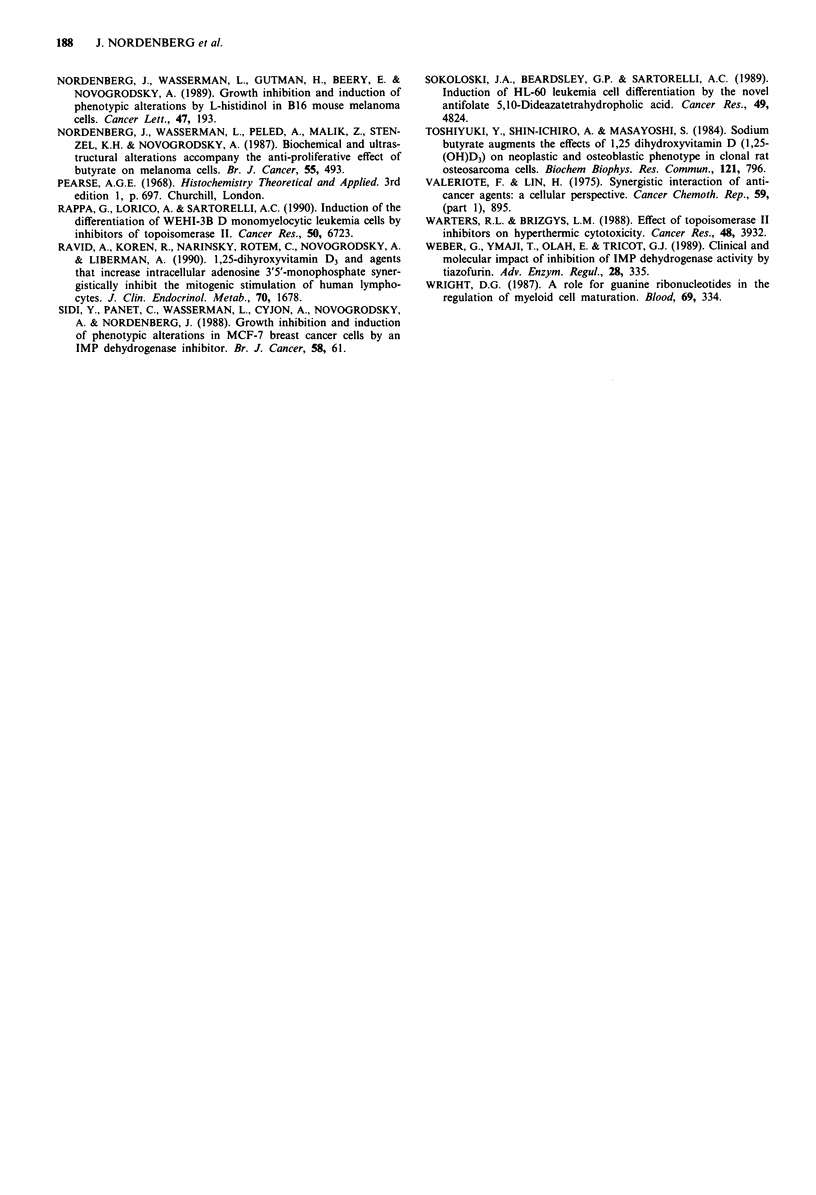# Novobiocin-induced anti-proliferative and differentiating effects in melanoma B16.

**DOI:** 10.1038/bjc.1992.38

**Published:** 1992-02

**Authors:** J. Nordenberg, D. Albukrek, T. Hadar, A. Fux, L. Wasserman, A. Novogrodsky, Y. Sidi

**Affiliations:** Endocrinology Laboratory, Beilinson Medical Center, Petah Tikva, Israel.

## Abstract

**Images:**


Br. J. Cancer (1992), 65, 183-188 © Macmillan Press Ltd., 1992

### Novobiocin-induced anti-proliferative and differentiating effects in melanoma B16

J. Nordenberg[1,2,3], D. Albukrek[3], T. Hadar[3,4], A. Fux[2,3], L. Wasserman[2], A. Novogrodsky[2,3] & Y. Sidi[3,5]

*FMRC and [2]Rogoff Research Institute, [1]Endocrinology Laboratory, [4]ENT and [5]Medicine D Departments, Beilinson Medical Center Petah Tikva 49100 and [3]Sackler School of Medicine Tel Aviv University, Ramat Aviv, Israel.*

**Summary** The antibiotic drug novobiocin was evaluated for its anti-tumour properties in B16 melanoma cells. Novobiocin is shown to inhibit melanoma B16 cell proliferation. The anti-proliferative effect was gradually reversible upon removal of novobiocin from the culture medium. Growth inhibition by novobiocin was accompanied by phenotypic alterations, that included morphological changes, lipid accumulation and marked increases in the activities of NADPH cytochrome c reductase and γ glutamyl transpeptidase. *In vivo* administration of repeated i.p. doses of novobiocin, to mice implanted with B16 melanoma cells resulted in growth retardation. The combined treatment of the B16 melanoma cells with novobiocin and other chemical inducers of differentiation was examined in a cell growth assay. Novobiocin and sodium butyrate inhibited cell growth in a near additive manner, while combination of novobiocin with the GTP-depleting agents, tiazofurin or mycophenolic acid resulted in a synergistic decrease in cell growth. Our results support the contention further that novobiocin and other differentiating agents might be of potential value in melanoma therapy.

Melanoma is known to be a chemotherapy-resistant cancer. Previous studies from our laboratory have focussed on the evaluation of the effects of chemical inducers of differentiation on mouse and human melanoma cells. These studies were aimed at the discovery of modalities that might modulate the cells to express a more differentiated and benign phenotype. We have shown that several agents, belonging to different chemical groups, such as dimethylsulfoxide, dimethylthiourea, sodium butyrate, histidinol and 8-hydroxyquinoline inhibit melanoma cell proliferation and induce differentiated characteristics in the cells (Nordenberg *et al.*, 1985; 1986; 1987; 1989; 1990). However, the compounds tested so far did not seem to be suitable for immediate clinical evaluation.

Novobiocin (Figure 1), a coumermycin antibiotic drug, which inhibits prokaryotic and eukaryotic DNA replication seemed to be an interesting candidate for evaluation as an anti-tumour differentiating agent in melanoma. The best known effect of novobiocin is its inhibition of topoisomerase II activity, due to interference with the ATPase subunits of the enzyme (Gellert, 1982). Novobiocin was recently shown to induce cell differentiation in several leukaemic cell lines (Constantinou *et al.*, 1989; Rappa *et al.*, 1990). Induction of differentiation in the HL-60 leukaemic cell line was attributed to inhibition of topoisomerase II activity (Constantinou *et al.*, 1989). Novobiocin was also reported to induce mitochondrial damage, leading to a decrease in the ATP/ADP ratio in Hela cells (Downes *et al.*, 1985). We have recently shown that inhibition of B16 melanoma cell growth and induction of differentiated features by dimethylthiourea was at least partially mediated by a decrease in ATP content (Fux *et al.*, 1991). Recent reports have also focussed interest on the anti-tumour properties of novobiocin. It was shown to exert *in vitro* and *in vivo* synergistic anti-tumour effects with alkylating agents (Eder *et al.*, 1987; 1989), to enhance cisplatinum cytoxicity (Eder *et al.*, 1987; 1988) and to increase sensitivity of ovarian cancer cells to hyperthermia (Warters *et al.*, 1988). Recently, a phase I trial of novobiocin in combination with cyclophosphamide has been conducted in patients with various disseminated malignancies (Eder *et al.*, 1991). Novobiocin was well tolerated in patients receiving cyclophosphamide and blood levels achieved were in the drug-potentiating range. The use of chemical inducers of differentiation in solid tumour cells might modify the cells in a way that renders them more susceptible to the effects of another differentiating agent. This was demonstrated by several investigators, using sodium butyrate in combination with 5'azacytidine, 1,25 dihydroxy vitamin D or retinoic acid (Jahangeer *et al.*, 1982; Toshiyuki *et al.*, 1984; Kyritsis *et al.*, 1985).

The aim of this study was to evaluate the effects of novobiocin on melanoma cell growth and differentiation and to examine its interaction with other differentiating agents.

### Materials and methods

Reagents for tissue culture were purchased from Biol. Industries, Novobiocin and reagents for enzyme assays were obtained from Sigma Chem. Comp.

C57/B1 mice (4-6 weeks old), fed ad libitum, from the animal unit of the Beilinson Medical Center were used.

### *Cell line*

B16 F10 murine melanoma cells were cultured in RPMI 1640, supplemented with 10% foetal calf serum, antibiotics and the anti-mycoplasma agent PPLO from Gibco Comp. as previously described (Nordenberg *et al.*, 1986). For growth experiments, cells ($4 \times 10^4$ 1.5 $ml^{-1}$) were incubated in tissue culture dishes (3 cm) with or without different concentrations of novobiocin. Cell number was determined by counting the cells in a Coulter counter following detachment of the cells with EDTA (1 mM).

**Figure 1** Chemical structure of novobiocin.

Correspondence: J. Nordenberg, Endocrinology Laboratory, Beilinson Medical Center, Petah-Tikva 49100, Israel.
Received 10 July 1991; and in revised form 31 October 1991.

*Determination of phenotypic alterations*

For demonstration of cell morphology and lipid droplets, cells were incubated in the presence and absence of novobiocin (100 μM) in tissue culture plates for 72 h. Cells were fixed with formol-calcium. Following fixation cells were stained by the oil red 0 method (Pearse, 1968) and visualised by light microscopy.

For determination of enzyme activities, cells ($7 \times 10^5$) in 10 ml of culture medium were incubated for 72 h in the absence or presence of novobiocin (100 μM). NADPH cytochrome c reductase and γ glutamyl transpeptidase activities were determined spectrophotometrically, as previously described (Nordenberg *et al.*, 1987). NADPH cytochrome c reductase activity was expressed as nmoles acceptor reduced $h^{-1}$ $mg^{-1}$ DNA.

γ Glutamyl transpeptidase activity was expressed as μmoles product formed $h^{-1}$ $mg^{-1}$ DNA. DNA was measured by the fluorometric method described by Labarca and Paigen (1980).

*Tumour cell inoculation and systemic administration of novobiocin*

$5 \times 10^4$ B16 melanoma cells were s.c. inoculated on the dorsum of the mice as previously described (Nordenberg *et al.*, 1985). Tumour growth was followed by measuring the tumour diameters in three dimensions, using calipers and tumour volume was calculated as previously described (Nordenberg *et al.*, 1985). 0.3 ml PBS with, or without novobiocin (150 mg $Kg^{-1}$) were injected i.p. according the following schedule: three times daily for the first 2 days after tumour cell inoculation and then twice daily for additional 10 days. (The number of daily novobiocin doses was decreased from three to two times daily because the mice suffered from diarrhoea).

*Preparation of short-term B16 cell cultures from tumour tissue*

Tumour tissue, which is encapsulated and very fluid was collected. Cells were disrupted mechanically under sterile conditions and filtered through gauze. The cell suspension was dissolved in RPMI 1640, containing 10% foetal calf serum and incubated in a humidified atmosphere at 37°C in a 5% $CO_2$ and 95% air atmosphere. After 48 h medium was replaced. 24–48 h later, the cells were detached and replated for additional 48 h and then used for growth experiments. It should be noted that the cells from the tumour tissue were heavily pigmented, while cells growing for many generations as a cell line in culture were poorly pigmented.

*Combined treatment of B16 cells with novobiocin and other differentiating agents*

Cells ($2 \times 10^4$) were incubated in 0.5 ml growth medium in multiwell plates (15 mm) plates for 72 h in the presence or absence of novobiocin, sodium butyrate, tiazofurin or mycophenolic acid alone or in combinations at the concentrations indicated in the figures. Cells were detached and counted as described above.

*Statistical analysis of data*

Paired *t*-test was used for evaluation of significance of the effects on enzyme activities and unpaired *t*-test was used for the *in vivo* studies.

The expected value for additive interaction of novobiocin with sodium butyrate or tiazofurin was calculated by using the formula described by Ravid *et al.* (1990) $F(A + B) = 1 - (1 - FA)(1 - FB)$ where FA and FB are the fractions of the proliferating cells inhibited by agents A and B. This calculation is based on the assumptions of Valeriote *et al.* (1975). The statistical significance of the synergistic interaction was assessed by the nonparametric sign test.

### Results

*Anti-proliferative effects of novobiocin* in vitro *and* in vivo

The effect of novobiocin on the proliferation of B16 melanoma cells grown for many generations in cell culture was examined by plating the cells in the absence and presence of various concentrations of novobiocin for 48 and 96 h. The number of untreated cells increased about 23 fold during the 96 h incubation period. Novobiocin induced a concentration dependent decrease in cell number as is shown in Figure 2. At 48 h 150 μM novobiocin decreased cell number beyond initial number plated, suggesting that this concentration led to an initial reduction in cell viability. However, proliferation of remaining cells did not stop as can be seen from the 96 h data. The cells that were counted after 96 h did not detach and were 95% viable as assessed by the trypan blue exclusion test.

Effects of differentiating agents on solid tumour cell lines are known to be reversible. In order to examine whether novobiocin-treated cells resume growth following removal of novobiocin from the culture medium cells were replated without novobiocin for different time periods. The results show that 4 days following removal of novobiocin from the medium, its growth inhibitory effect was still maintained (Figure 3a). However, growing the cells for 11 days in novobiocin-free medium, resulted in restoration of normal growth rate (Figure 3b).

The sensitivity of cells to growth-inhibition by novobiocin in short term cultures prepared from fresh melanoma tumours was compared to that of the cells grown as a continuous cell line. Both cell types were found to be equally sensitive to growth inhibition by novobiocin (Results not shown). This finding encouraged us to examine the effect of *in vivo* application of novobiocin to mice inoculated with B16 melanoma cells. Mice inoculated s.c. with $5 \times 10^4$ viable B16 cells developed visible spherical tumours on their dorsum within 10–12 days after inoculation, leading to death of the mice within 20–30 days. The results depicted in Figure 4 show that repeated daily i.p. injections of novobiocin resulted in delayed tumour growth in the novobiocin-treated group. Measurements of tumour volume were stopped on day 20,

**Figure 2** The effect of novobiocin on B16 F10 melanoma cell proliferation. Cells were incubated and counted as described in methods. Values are means ± s.d. for three plates for each concentration.

since three out of eight mice in the untreated group were already dead at this time. Less aggressive treatment schedules, for example application of single daily injection of novobiocin for 5 days, were tried but failed to induce a delay in tumour growth.

**Figure 3** Cell growth of B16 melanoma following removal of novobiocin from the medium. **a**, $2 \times 10^5$ untreated or novobiocin pre-treated (72 h) cells were incubated in 5 ml novobiocin-free medium for 96 h. Cells were detached and counted as described in the methods. **b**, Untreated or novobiocin pre-treated (72 h) cells were grown in novobiocin-free medium for 7 days. Cells were replated at $2 \times 10^5$ cells in 5 ml novobiocin free-medium, for additional 96 h. Cells were detached and counted as described in the methods.

**Figure 4** *In vivo* anti-tumour activity of novobiocin. Inoculation of cells and calculation of tumour volumes was described in methods. Values are mean volumes ± s.e. for eight mice in each group. Untreated *vs* novobiocin-treated at days 16 and 18 ($P<0.01$) and at day 20 ($P<0.001$).

### *Phenotypic alterations induced by novobiocin*

The anti-proliferative effects of novobiocin were accompanied by phenotypic alterations, that resembled those induced by other chemical inducers of differentiation. Novobiocin altered cell morphology. The untreated cells were spindle shaped, whereas the treated cells were flat, spread with elongated appendages and seemed to be enlarged. Novobiocin-treatment induced the accumulation of lipid droplets in the cytoplasm (Figure 5). Incubation of the B16 cells with novobiocin for 72 h resulted in a marked enhancement of the activities of the plasma membrane-bound enzyme γ glutamyl transpeptidase and of the endoplasmic reticulum marker enzyme NADPH cytochrome c reductase (Table I).

### *Combined anti-proliferative effects of novobiocin and other differentiating agents*

The effect on cell growth of combined treatment of B16 cells with novobiocin and other chemical inducers of differentiation was examined. Incubation of the B16 cells

**Figure 5** Morphological appearance and lipid content of untreated and novobiocin-treated B16 F10 cells. **a**, Untreated cells × 200. **b**, 72 h Novobiocin (100 μM)-treated cells. × 200.

**Table I** The effect of novobiocin on the activities of NADPH cytochrome c reductase and γ glutamyl transpeptidase

| *Treatment* | *NADPH cytochrome c reductase nmoles mgDNA⁻¹ h⁻¹* | *γ glutamyl transpeptidase μmoles mgDNA⁻¹ h⁻¹* |
|---|---|---|
| Untreated | 6.18 ± 0.12 | 4.82 ± 0.19 |
| Novobiocin (100 μM) | 22.90 ± 0.66[a] | 27.62 ± 1.24[a] |

Values are means ± s.e. of five independent experiments. [a]$P<0.001$.

with sodium butyrate at a dose leading to a 40% decrease in cell number in combination with several concentrations of novobiocin resulted in a near additive growth inhibitory effect (Figure 6).

The effect of combined treatment of the B16 cells with novobiocin and either tiazofurin or mycophenolic acid is depicted in Figures 7 and 8. The combination of novobiocin with mycophenolic acid, or with tiazofurin resulted in a decrease in cell number that was greater than the expected calculated value of an additive interaction, suggesting that novobiocin and GTP-depleting agents may interact synergistically.

### Discussion

Novobiocin was shown to induce anti-proliferative effects *in vitro* and *in vivo* in B16 melanoma. Growth inhibition by novobiocin was reversible and was accompanied by phenotypic alterations that resemble those induced by other differentiating agents. These included morphological changes, lipid droplet accumulation and enhancement of the activities of NADPH cytochrome c reductase and glutamyl transpeptidase. We have previously shown that this specific pattern of phenotypic alterations was induced by the well known chemical inducer of differentiation sodium butyrate in mouse and human melanoma cell lines (Nordenberg *et al*., 1986; 1987). As previously suggested, the phenotypic alterations induced by the differentiating agents are at least in part compatible with a more differentiated state. The increase in NADPH cytochrome c reductase, a marker enzyme of the endoplasmic reticulum has been described to accompany the action of all chemical inducers of differentiation that produced anti-proliferative effects in melanoma cells (Fux *et al*., 1989). The development of endoplasmic reticulum and the increase in its marker enzyme (NADPH cytochrome c reductase) might reflect cell differentiation, as normal melanocytic development is also accompanied by the appearance of endoplasmic reticulum and golgi complexes (Jimbo & Vesugi, 1982). A potential role for γ glutamyl transpeptidase in the synthesis of pheomelanin from 5-S-cysteinyldopa has been suggested (Mojamdar *et al*., 1983). Although novobiocin did not induce a change in pigmentation similar to sodium butyrate, a marked increase in the activity of this enzyme was observed. Other melanoma differentiating agents, such as α MSH and theophylline were also reported to enhance the activity of this enzyme (Hu, 1982; Mojamdar *et al*., 1983). In leukemic HL-60 cells, the differentiating effect of novobiocin was associated with a reduction in topoisomerase II activity (Constantinou *et al*., 1989). Although similar concentrations of novobiocin were effective on the melanoma cells, further studies are required to link the anti-proliferative and differentiating effects in the melanoma cells with decreased

Figure 7 Combined effects of novobiocin and tiazofurin on B16 melanoma cell growth. Cells ($2 \times 10^4 \times 0.5\,ml^{-1}$) were incubated for 72 h in the absence and presence of different tiazofurin and novobiocin concentrations, alone and in combinations (A,B). Values are means ± s.e. for 6–9 replicates done with different cell preparations. The expected values for an additive interaction were calculated as described in the Methods section. The difference between the expected and obtained values was significant $P<0.01$.

Figure 8 Combined effects of novobiocin and mycophenolic acid on B16 melanoma cell growth. Cells ($2 \times 10^4 \times 0.5\,ml^{-1}$) were incubated for 72 h in the absence and presence of different concentrations of mycophenolic acid and novobiocin, alone and in combination. Values are means of 3–5 replicates ± s.d. of one out of two experiments done with different cell preparations. The expected values of an additive interaction were calculated as described in the Methods section.

Figure 6 The effect of combined addition of novobiocin and sodium butyrate on B16 melanoma cell growth. Cells ($4 \times 10^4 \times ml^{-1}$) were incubated in the absence and presence of sodium butyrate alone, novobiocin alone, or novobiocin + sodium butyrate for 72 h. Values are means of six replicates done with different cell preparations ± s.e. The expected values for an additive interaction were calculated as described in the Methods section.

topoisomerase II activity. Recent results obtained in our laboratory indicate that novobiocin induces a moderate decrease in ATP content of the cells (Novobiocin at 0.075 mM induced a decrease in intracellular ATP levels of about 30%. The ATP content of untreated cells was $497 \pm 57$ nmoles mg$^{-1}$ DNA and of 0.075 mM novobiocin-treated cells was $357 \pm 39$ nmoles mg$^{-1}$ DNA). It is possible that ATP depletion is related to the appearance of a more differentiated phenotype. This suggestion is supported by our previous findings (Fux *et al.*, 1991).

Several combinations of differentiating agents with sodium butyrate resulted in synergistic interactions. Novobiocin and sodium butyrate were reported to have synergistic effects on transformation of Chang liver cell into fibroblast-like cells (Kaneko *et al.*, 1988). These authors also found that novobiocin, similar to sodium butyrate increased nuclear protein acetylation. The combined treatment of the B16 cells with novobiocin and sodium butyrate did not result in a more than additive interaction. This may suggest that these agents act either independently on the cells, or by the same pathway.

GTP-depleting agents, such as mycophenolic acid and tiazofurin were shown to be inducers of cell differentiation in several cell types, including melanoma cells (Wright, 1987; Sidi *et al.*, 1988). Tiazofurin was also used in clinical trials (Weber *et al.*, 1989).

The mechanism for the combined synergistic interaction of novobiocin and GTP-depleting agents has not been explored so far. However, the decrease in both ATP and GTP might provide a clue to the interaction of novobiocin and the GTP-depleting agents. A methotrexate analog that depletes ATP and GTP content has recently been reported to be a potent inducer of leukaemic cell differentiation (Sokoloski *et al.*, 1990).

Previous studies of Eder *et al.* (1987; 1989) demonstrated enhancement of the anti-tumour effects of alkylating agents in leukaemia and fibrosarcoma bearing mice. In this study an *in vivo* growth inhibitory effect of novobiocin was found in melanoma bearing mice.

The mechanism of *in vivo* growth inhibition by novobiocin is as yet unclear. The multiple targets that have been described for the action of novobiocin (Gellert, 1982; Downes *et al.*, 1985; Lynch *et al.*, 1976; Edenberg, 1980), do not identify a specific mechanism. However, our data showing induction of differentiation by novobiocin *in vitro* may imply that phenotypic alterations occurring *in vivo* contribute to the observed anti-tumour effect. The present study encourages further investigation of the mechanism of the phenotypic alterations induced by novobiocin in melanoma.

Novobiocin has a profound advantage over other differentiating agents used so far, since it is a drug already used as anti-microbial chemotherapy (Drusano *et al.*, 1986). This fact obviously facilitated the phase I trial with novobiocin for solid tumours, including melanoma, that has recently been reported (Eder *et al.*, 1991).

This study was partially supported by the Schreiber Fund, Tel-Aviv University.

Tiazofurin was kindly provided as a gift by the Drug Synthesis and Chemistry Branch Division of Cancer Treatment, National Cancer Institute, Bethesda, MA, USA.

### References

CONSTANTINOU, A., HENNING-CHUBB, C. & HUBERMAN, E. (1989). Novobiocin-and phorbol-12-myristate-13-acetate-induced differentiation of human leukemia cells associated with a reduction in topoisomerase II activity. *Cancer Res.*, **49**, 1100.

DOWNES, C.S., ORD, M.J., MULLINGER, A.M. & COLLINS, A.R.S. & JOHNSON, R.T. (1985). Novobiocin inhibition of DNA excision repair may occur through effects on mitochondrial structure and ATP metabolism, not on repair topoisomerases. *Carcinogenesis*, **6**, 1343.

DRUSANO, G.L., TOWNSEND, R.J., WALSH, T.J., FOREST, A., ANTAL, E.J. & STANDIFORD, H.C. (1986). Steady state serum pharmacokinetics of novobiocin and rifampin alone and in combination. *Antimicrob. Agents. Chemother.*, **30**, 42.

EDER, J.P., TEICHER, B.A., HOLDEN, S.A., CATHCART, K.N.S. & SCHNIPPER, L.E. (1987). Novobiocin enhances alkylating agent cytotoxicity and DNA interstrand crosslinks in a murine model. *J. Clin. Invest.*, **79**, 1524.

EDER, J.P., TEICHER, B.A., HOLDEN, S.A., CATHCART, K.N., FREI, E. & SCHNIPPER, L.S. (1988). Novobiocin enhancement of cisplatin cytotoxicity is mediated by DNA topoisomerase II. *Proc. Ann. Meeting Am. Assoc. Cancer Res.*, **29**, 1056.

EDER, J.P., TEICHER, B.A., HOLDEN, S.A., CATHCART, K.N., SCHNIPPER, L.E. & FREI, E. (1989). Effect of novobiocin on the antitumor activity and tumor cell and bone marrow survivals of three alkylating agents. *Cancer Res.*, **49**, 595.

EDER, J.P., WHEELER, C.A., TEICHER, B.A. & SCHNIPPER, L.E. (1991). A phase I clinical trial of novobiocin, a modulator of alkylating agent cytotoxicity. *Cancer Res.*, **51**, 510.

EDENBERG, H.J. (1980). Novobiocin inhibition of simian virus 40DNA replication. *Nature*, **286**, 529.

FUX, A., NORDENBERG, J., WASSERMAN, L., MALIK, Z., PELED, A. & NOVOGRODSKY, A. (1989). Increased NADPH cytochrome c reductase activity – a marker for the action of chemical inducers of differentiation on melanoma cells. In *Advances in Animal Biology and Technology for Bioprocesses*. Spier, R.E., Griffiths, J.B., Stephenne, J. & Crooy, P.J. (eds), p. 175, Butterworths: UK.

FUX, A., SIDI, Y., KESSLER-ICEKSON, G., WASSERMAN, L., NOVOGRODSKY, A. & NORDENBERG, J. (1991). Dimethylthiourea, inhibition of B16 melanoma growth and induction of phenotypic alterations; relationship to ATP levels. *Br. J. Cancer*, **63**, 489.

GELLERT, M. (1982). DNA topoisomerases. *Ann. Rev. Biochem.*, **59**, 879.

HU, F. (1982). Theophylline and melanocyte stimulating hormone effects on gamma glutamyl transpeptidase and DOPA reactions in cultured melanoma cells. *J. Invest. Dermatol.*, **79**, 57.

JAHANGEER, S., ELLIOTT, R.M. & HENNEBERRY, C. (1982). β adrenergic receptor induction in hela cells: synergistic effect of 5-azacytidine and butyrate. *Biochem. Biophys. Res. Commun.*, **108**, 1434.

JIMBO, K. & VESUGI, T. (1982). New melanogenesis and photobiological processes in activation and proliferation of precursor melanocytes after UV-exposure: Ultrastructural differentiation of precursor melanocytes from Langerhans cells. *J. Invest. Dermatol.*, **78**, 108.

KANEKO, Y., NAKAYAMA, T., HAMASAKI, T., TSUKAMOTO, A., TODA, G. & OKA, H. (1988). Fibroblast-like transformation and c-*myc* gene alteration of human hepatocytes induced by novobiocin and butyrate. *Biochem. Biophys. Res. Comm.*, **155**, 305.

KYRITSIS, A.P., WIGGERT, B., LEE, L. & CHADER, G.J. (1985). Butyrate enhances the synthesis of interphotoreceptor retinoid binding protein (IRBP) by Y-79 human retinoblastoma cells. *J. Cell. Physiol.*, **124**, 233.

LABARCA, C. & PAIGEN, K. (1980). A simple, rapid and sensitive DNA assay procedure. *Anal. Biochem.*, **102**, 344.

MOJAMDAR, M., ICHIHASHI, M. & MISHIMA, Y. (1983). Glutamyl transpeptidase, tyrosinase and 5-S-cysteinyldopa production in melanoma cells. *J. Invest. Dermatol.*, **81**, 119.

LYNCH, W.E., SHORT, J. & LIEBERMAN, J. (1976). The 7.1S nuclear DNA polymerase and DNA replication in intact liver. *Cancer Res.*, **36**, 901.

NORDENBERG, J., ALONI, D., WASSERMAN, L., BEERY, E., STENZEL, K.H. & NOVOGRODSKY, A. (1985). Dimethylthiourea inhibition of melanoma cell growth *in vitro* and *in vivo*. *J. Natl Cancer, Inst.*, **75**, 891.

NORDENBERG, J., NOVOGRODSKY, A., BEERY, E., PATIA, M., WASSERMAN, L. & WARSHAWSKY, A. (1990). Antiproliferative effects and phenotypic alterations induced by 8-hydroxyquinoline in melanoma cell lines. *Eur. J. Cancer*, **26**, 905.

NORDENBERG, J., WASSERMAN, L., BEERY, E. & 4 others (1986). Growth inhibition of murine melanoma by butyric acid and dimethylsulfoxide. *Exp. Cell Res.*, **162**, 77.

NORDENBERG, J., WASSERMAN, L., GUTMAN, H., BEERY, E. & NOVOGRODSKY, A. (1989). Growth inhibition and induction of phenotypic alterations by L-histidinol in B16 mouse melanoma cells. *Cancer Lett.*, **47**, 193.

NORDENBERG, J., WASSERMAN, L., PELED, A., MALIK, Z., STENZEL, K.H. & NOVOGRODSKY, A. (1987). Biochemical and ultrastructural alterations accompany the anti-proliferative effect of butyrate on melanoma cells. *Br. J. Cancer*, **55**, 493.

PEARSE, A.G.E. (1968). *Histochemistry Theoretical and Applied*. 3rd edition 1, p. 697. Churchill, London.

RAPPA, G., LORICO, A. & SARTORELLI, A.C. (1990). Induction of the differentiation of WEHI-3B D monomyelocytic leukemia cells by inhibitors of topoisomerase II. *Cancer Res.*, **50**, 6723.

RAVID, A., KOREN, R., NARINSKY, ROTEM, C., NOVOGRODSKY, A. & LIBERMAN, A. (1990). 1,25-dihyroxyvitamin $D_3$ and agents that increase intracellular adenosine 3′5′-monophosphate synergistically inhibit the mitogenic stimulation of human lymphocytes. *J. Clin. Endocrinol. Metab.*, **70**, 1678.

SIDI, Y., PANET, C., WASSERMAN, L., CYJON, A., NOVOGRODSKY, A. & NORDENBERG, J. (1988). Growth inhibition and induction of phenotypic alterations in MCF-7 breast cancer cells by an IMP dehydrogenase inhibitor. *Br. J. Cancer*, **58**, 61.

SOKOLOSKI, J.A., BEARDSLEY, G.P. & SARTORELLI, A.C. (1989). Induction of HL-60 leukemia cell differentiation by the novel antifolate 5,10-Dideazatetrahydropholic acid. *Cancer Res.*, **49**, 4824.

TOSHIYUKI, Y., SHIN-ICHIRO, A. & MASAYOSHI, S. (1984). Sodium butyrate augments the effects of 1,25 dihydroxyvitamin D (1,25-$(OH)D_3$) on neoplastic and osteoblastic phenotype in clonal rat osteosarcoma cells. *Biochem Biophys. Res. Commun.*, **121**, 796.

VALERIOTE, F. & LIN, H. (1975). Synergistic interaction of anticancer agents: a cellular perspective. *Cancer Chemoth. Rep.*, **59**, (part 1), 895.

WARTERS, R.L. & BRIZGYS, L.M. (1988). Effect of topoisomerase II inhibitors on hyperthermic cytotoxicity. *Cancer Res.*, **48**, 3932.

WEBER, G., YMAJI, T., OLAH, E. & TRICOT, G.J. (1989). Clinical and molecular impact of inhibition of IMP dehydrogenase activity by tiazofurin. *Adv. Enzym. Regul.*, **28**, 335.

WRIGHT, D.G. (1987). A role for guanine ribonucleotides in the regulation of myeloid cell maturation. *Blood*, **69**, 334.